# Continuous Cell Lines from the European Biting Midge *Culicoides nubeculosus* (Meigen, 1830)

**DOI:** 10.3390/microorganisms8060825

**Published:** 2020-05-30

**Authors:** Lesley Bell-Sakyi, Fauziah Mohd Jaafar, Baptiste Monsion, Lisa Luu, Eric Denison, Simon Carpenter, Houssam Attoui, Peter P. C. Mertens

**Affiliations:** 1 Institute of Infection, Veterinary and Ecological Sciences, University of Liverpool, 146 Brownlow Hill, Liverpool L3 5RF, UK; lisaluu@liverpool.ac.uk; 2 UMR1161 Virologie, INRAE, Ecole Nationale Vétérinaire d’Alfort, ANSES, Université Paris-Est, 94700 Maisons-Alfort, France; fauziah.mohd-jaafar@vet-alfort.fr (F.M.J.); baptiste.monsion@vet-alfort.fr (B.M.); houssam.attoui@vet-alfort.fr (H.A.); 3 The Pirbright Institute, Ash Road, Pirbright, Woking, Surrey GU24 0NF, UK; eric.denison@pirbright.ac.uk (E.D.); simon.carpenter@pirbright.ac.uk (S.C.); 4 School of Veterinary Medicine and Science, University of Nottingham, Sutton Bonington Campus, Sutton Bonington LE12 5RD, UK; Peter.Mertens@nottingham.ac.uk

**Keywords:** *Culicoides*, Ceratopogonidae, *Monoculicoides*, cell line, vector, midge, bluetongue virus, orbivirus, virus replication

## Abstract

*Culicoides* biting midges (Diptera: Ceratopogonidae) transmit arboviruses of veterinary or medical importance, including bluetongue virus (BTV) and Schmallenberg virus, as well as causing severe irritation to livestock and humans. Arthropod cell lines are essential laboratory research tools for the isolation and propagation of vector-borne pathogens and the investigation of host-vector-pathogen interactions. Here we report the establishment of two continuous cell lines, CNE/LULS44 and CNE/LULS47, from embryos of *Culicoides nubeculosus*, a midge distributed throughout the Western Palearctic region. Species origin of the cultured cells was confirmed by polymerase chain reaction (PCR) amplification and sequencing of a fragment of the *cytochrome oxidase* *1* gene, and the absence of bacterial contamination was confirmed by bacterial 16S rRNA PCR. Both lines have been successfully cryopreserved and resuscitated. The majority of cells examined in both lines had the expected diploid chromosome number of 2*n* = 6. Transmission electron microscopy of CNE/LULS44 cells revealed the presence of large mitochondria within cells of a diverse population, while arrays of virus-like particles were not seen. CNE/LULS44 cells supported replication of a strain of BTV serotype 1, but not of a strain of serotype 26 which is not known to be insect-transmitted. These new cell lines will expand the scope of research on *Culicoides*-borne pathogens.

## 1. Introduction

Biting midges of the genus *Culicoides* are vectors of a variety of pathogens of veterinary or medical importance, including viruses, protozoa and helminths [1,2,3,4,5]. In Europe, *Culicoides* midges transmit bluetongue virus (BTV) and Schmallenberg virus (SBV) that cause severe, economically important diseases in ruminants [6,7,8,9,10,11]. In addition, they have acted as vectors of African horse sickness virus (AHSV), which causes one of the most lethal known diseases of horses, during sporadic outbreaks in Spain and Portugal [12]. *Culicoides* were also recognised over 40 years ago as being implicated in the debilitating skin condition “sweet itch” in horses [13], and cause severe irritation to humans through their bites [14].

The European midge species *Culicoides nubeculosus* (Meigen 1830), which is found across the Western Palearctic region from Spain and the United Kingdom in the west to Poland and Turkey in the east [15,16,17,18], is considered to have a low vectorial capacity for BTV and SBV [10,11]. *C. nubeculosus* transmits the filarial worm *Onchocerca cervicalis* [5], and coinfecting *O. cervicalis* were found to increase susceptibility to BTV in a small proportion of midges [19]. *C. nubeculosus* is one of very few species of the Ceratopogonidae that has been maintained continuously in colonies, although only one primary line, that was established from a field population in 1969 [16], is currently extant. The subgenus *Monoculicoides*, to which *C. nubeculosus* belongs, is notable for larvae that possess heavily sclerotised mouthparts that enable an omnivorous diet and this may facilitate colonisation, as two other species within this subgenus, *Culicoides sonorensis* Wirth and Jones and *Culicoides riethi* Kieffer, have been colonised and maintained successfully for several years [20]. Other species of *Culicoides* in Europe including *Culicoides imicola* Kieffer, the major sub-Saharan vector of BTV and AHSV, and *Culicoides impunctatus* Goetghebuer, the species inflicting the most severe biting nuisance on humans, have been more challenging to maintain in the laboratory, often refusing to mate and with slow developmental times and high rates of mortality across life stages [20].

Continuous cell lines derived from arthropods such as mosquitoes and ticks are essential laboratory research tools for isolation and propagation of vector-borne pathogens and investigation of host-vector-pathogen interactions [21]. Biting midge cell lines derived from the North American species *C. sonorensis*, previously known as *Culicoides variipennis* [22,23,24], have been used in studies on orbivirus replication and transmission [25,26,27,28], *Culicoides* antiviral immunity [29] and ability to support infection and growth of the bacterial symbiont *Wolbachia* [30]. One of these lines, KC, is used routinely in reference laboratories to isolate emerging arbovirus strains, in particular of BTV. However, no cell lines have been developed from Old World *Culicoides* species, and the existing *C. sonorensis* cell lines may have limited applicability to research on arboviruses and other microorganisms prevalent in the European environment.

Here we report the establishment and partial morphological and molecular characterisation of two continuous cell lines from embryos of the European midge species *C. nubeculosus*. The ability of one of the cell lines was tested to support the replication of strains of two serotypes of BTV, BTV-1 known to be transmitted by midges and BTV-26 with no known arthropod vector.

## 2. Materials and Methods 

### 2.1. Generation of Primary C. nubeculosus Cultures

*C. nubeculosus* eggs used in the study were produced at The Pirbright Institute as described previously [31], except that a Hemotek blood feeder (Hemotek, Blackburn, UK), was used to feed adult females on commercially-supplied horse blood (TCS Biosciences, Buckingham, UK). The colony was initiated in 1969 from adult *C. nubeculosus* midges collected in Hertfordshire, UK [16] and has been maintained continuously since, as a closed colony. Eggs laid on damp filter paper within the preceding 24 h were transported to the Tick Cell Biobank (maintenance temperature range between 4 °C and 22 °C) where they were incubated at 4 °C or 15 °C for 0–5 days prior to processing. 

Primary cultures were prepared following the method used originally with *C. sonorensis* eggs [22] with some modifications. The eggs were detached from the filter paper in an 0.1% aqueous solution of benzalkonium chloride and soaked for 10 min; clumps of eggs were broken up by pipetting. The egg suspension was then centrifuged at 1050× *g* for 2 min, the supernate was removed and the eggs resuspended in 70% ethanol. After 5 min the egg suspension was centrifuged again, the ethanol replaced with Hanks balanced salt solution (HBSS), and the egg suspension centrifuged again. Finally, the eggs were resuspended in 0.5 mL of HBSS or complete culture medium, transferred to a 35 mm sterile plastic petri dish and crushed with the flattened end of a sterile glass rod. The resultant suspension of midge tissues, eggshells and some uncrushed eggs was diluted with additional HBSS or complete medium in a centrifuge tube and allowed to settle for about 2 min. The topmost layer of settled material was collected in about 0.5 mL and transferred to a flat-sided culture tube (Nunc, Thermo-Fisher, Loughborough, UK) to which was added a further 1.5 mL of complete culture medium. All medium components were obtained from Invitrogen (Thermo-Fisher, Loughborough, UK) or Sigma (Gillingham, UK) unless otherwise indicated. The complete media used were: L-15 (Leibovitz) supplemented with 10% tryptose phosphate broth (TPB), 20% foetal bovine serum (FBS), 2mM L-glutamine (L-glut), 100 units/mL penicillin and 100 µg/mL streptomycin (pen/strep) (L-15); HBSS supplemented with 0.5% lactalbumin hydrolysate, 20% FBS, L-glut and pen/strep (H-Lac); L-15B [32] supplemented with 10% TPB, 10% FBS, L-glut, pen/strep and 0.1% bovine lipoprotein concentrate (MP Biomedicals, Thermo-Fisher, Loughborough, UK) (L-15B); Schneider’s modified *Drosophila* medium (Lonza, Nottingham, UK) supplemented with 10% FBS, L-glut and pen/strep (Schneider’s). Cultures were incubated at 28 °C in sealed containers in ambient air in a dry incubator. The medium was changed weekly by removal and replacement of 1.0–1.5 ml medium. Cultures were examined weekly with an inverted microscope for signs of tissue viability and cell growth.

### 2.2. Establishment of C. nubeculosus Cell Lines

When inverted microscope examination revealed the occurrence of significant levels of cell multiplication, characterised by the formation of floating clumps of multicellular vesicles and patches of attached small elongated, spindle-shaped or epithelial-like cells, subculture was carried out. After the medium change, an equal volume of fresh medium was added to a primary culture, the attached tissue clumps and cells were pipetted off, mixed gently and half of the cell suspension was transferred to a new flat-sided tube, leaving the remainder in the parent tube. When the daughter tube showed evidence of further cell growth, the procedure was repeated.

When sufficient numbers of daughter tubes were available, the contents of 3–4 tubes were pooled and centrifuged at 400× *g* for 5 min, the supernate was discarded, the cell pellet was resuspended in 1.5–2 mL of ice-cold complete medium with 20% FCS, an equal volume of ice-cold complete medium with 20% FCS and 20% dimethyl sulphoxide (DMSO) was added and the cell suspension was immediately divided in 1 mL aliquots into ice-cold cryovials. The cryovials were frozen rapidly in dry ice and transferred within 30 min to the vapour phase of a liquid nitrogen refrigerator. Cryopreserved cells were resuscitated by rapidly thawing the cryovial in a 37 °C water bath, immediately transferring the contents to 9 mL complete medium with 20% FCS at room temperature, centrifuging at 400× *g* for 5 min, resuspending the pellet in 2.2 mL of medium with 20% FCS, transferring to a sealed flat-sided tube and incubating at 28 °C. 

### 2.3. Characterisation of C. nubeculosus Cell Lines CNE/LULS44 and CNE/LULS47

Cytocentrifuge smears were prepared from both cell lines using a Cytospin 3 (Shandon Southern, Runcorn, UK), air-dried, fixed for 3 min in methanol and stained for 20 min in 10% Giemsa stain. DNA was extracted from both cell lines using a DNeasy Mini Kit (Qiagen, Manchester, UK) following the manufacturer’s instructions for cultured cells. A 710 bp fragment of the mitochondrial *cytochrome oxidase 1* (*cox1*) gene was amplified following a previously published protocol [33]. A pan-bacterial PCR targeting a 1500 bp fragment of the bacterial 16S rRNA gene [34] was used to screen the cell lines for presence of endosymbiotic or contaminating bacteria. Both cell lines were screened for contaminating *Mycoplasma* using the Myco-Alert kit (Lonza, Nottingham, UK) and the *Mycoplasma* PCR ELISA kit (Roche Diagnostics, Newhaven, UK), following the manufacturers’ instructions.

Chromosome spreads were prepared from both cell lines as follows. Cells were seeded into flat-sided tubes and incubated at 28 °C for 44 h; 625 μL of colcemid (10 µg/mL, Roche Diagnostics, Newhaven, UK) was added to arrest cell development at metaphase, and the cells were incubated for a further 18 h. Cells were then harvested, centrifuged at 400× *g* for 5 min and the pellet re-suspended in 5 mL 0.75% sodium citrate and incubated for 35 min at 37 °C. The cell lysate was centrifuged as before, the pellet was re-suspended in 5 mL ice-cold acetic alcohol (1 part glacial acetic acid:3 parts technical methanol) and held on ice for 5 min. This process was repeated, and finally, the cell lysate was centrifuged as before and the pellet was gently resuspended in an equal volume of ice-cold acetic alcohol. Single drops were dripped onto ice-cold, wet, clean microscope slides from a height of approximately 90 cm. The slides were air-dried and then stained with 3% Giemsa for 1 h. The slides were examined under a light microscope at a magnification of × 250 to count the chromosomes in at least 100 spreads per cell line.

Cell line CNE/LULS44 was prepared for transmission electron microscopy (TEM) as follows. Cells were harvested, centrifuged at 1000× *g* for 5 min, washed once in PBS and fixed in 2.5% glutaraldehyde (*w*/*v*) in 0.1 M phosphate buffer (0.08M Na_2_HPO_4_, 0.02M NaH_2_PO4, pH 7.4) in a Pelco Biowave (Ted Pella Inc., Redding, CA, USA). The cells were then washed three times in 0.1M phosphate buffer before being embedded in 3% agarose, set on ice and cut into small cubes. The cubes were then post-fixed and stained with 2% aqueous osmium tetroxide in the Pelco Biowave, followed by 2% aqueous uranyl acetate overnight at 4 °C. To prevent precipitation artefacts, the cubes were washed for a minimum of 5 × 3 min with double-distilled water (ddH_2_O) between each staining step. The cubes were then washed in ddH_2_O before dehydration in a graded series of acetone at 30%, 50%, 70% and 90% in ddH_2_O for 5 min each, followed by 2 × 5 min in 100% acetone. Samples were then infiltrated with medium Premix resin (TAAB, Aldermaston, UK) at 30%, 50% and 75% resin in acetone for 30 min each, followed by 3 × 100% resin steps for 30 min each. Fresh 100% resin was used to embed pellets in silicone moulds before being cured for 48 h at 60 °C. Ultrathin serial sections (70–75 nm) were cut on a UC6 ultramicrotome (Leica Microsystems, Milton Keynes, UK) and collected on formvar coated copper grids. Grids were post-stained with 4% uranyl acetate and lead citrate, before viewing at 120KV in an FEI Tecnai G2 Spirit transmission electron microscope (FEI, Thermo-Fisher, Loughborough, UK). Images were taken with a RIO16 camera (Gatan Inc., Leicester, UK) using GMS3 software.

### 2.4. Infection of C. nubeculosus Cell Line CNE/LULS44 with BTV

CNE/LULS44 cells were seeded in 24-well plates at a density of approximately 5 × 10^5^ cells per well and incubated for 48 h at 28 °C inside a humidified container. *C. sonorensis*-derived KC cells [23], maintained in Schneider’s medium supplemented as above and treated identically to the *C. nubeculosus* cells, were used as controls. Triplicate wells were infected with ~4 × 10^4^ plaque-forming units (PFU) per well of BTV-1 (strain RSArrrr/01 [35]) or BTV-26 (strain KUW2010/02 [28]), and samples collected on days 0, 4, 9 and 14 post-infection (p.i.). The contents of each well were resuspended and centrifuged at 2000× *g* for 10 min at 4 °C to separate cells and supernates. RNA was extracted using RNA NOW (Biogentex, Houston, TX, USA)) as described previously [36]. Briefly, each cell pellet was dissolved in 1 mL of RNA NOW reagent and RNA was purified separately from cells and supernates following the manufacturer’s instructions. Real-time RT-PCRs were performed on equal volumes of RNA pooled for each fraction from the triplicate wells for each time point using primers/probe specific for BTV segment 10 (BTV_S10_F: TGGAYAAAGCRATGTCAAA, BTV_S10_P: FAM-ARGCTGCATTCGCATCGTACGC-BHQ1, BTV_S10_R: ACRTCATCACGAAACGCTTC) and cycling conditions as described previously [37]. PFU equivalents were calculated from cycle threshold (Ct) values as follows. Two 75 cm^2^ flasks of BSR cells [38] inoculated with BTV-1 were harvested at 72 h p.i. The cell pellet was collected by centrifugation at 2000× *g* for 10 min. The supernate was discarded and the pellet was suspended in 5 mL of Glasgow’s modified Eagle’s medium (GMEM), treated with an equal volume of Vertrel XF (Sigma, St Quentin Fallavier, France) as previously described [39], then treated with RNase-A (Roche, Indianapolis, IN, USA) and benzonase (Novagen, Madison, WI, USA) to remove non-encapsidated nucleic acids. The volume of the aqueous phase was made up to 40 mL by adding GMEM. Ten-fold serial dilutions of the purified virus suspension were prepared in serum-free GMEM, for titration in triplicate by plaque reduction neutralisation test [40]. RNA was also extracted from the same serial dilutions, using RNA NOW. A standard curve was generated to compare Ct values from real-time RT-PCR assays with virus titres (PFU/mL) for BTV-1. The curve showed a high correlation (R^2^ = 0.982). The number of PFU-equivalents for BTV-1 were calculated using the formula: y = −1.916ln(x) + 40.776, where y is the Ct value determined by real-time RT-PCR assay and x is the number of PFU-equivalents/mL. For BTV-1, the value of x was x = e^(y−40.776)/(−1.916)^, where e stands for exponential, the mathematical constant or base of the natural logarithm that equals 2.71828. 

## 3. Results

### 3.1. Generation of C. nubeculosus Cell Lines

**Figure 1:**
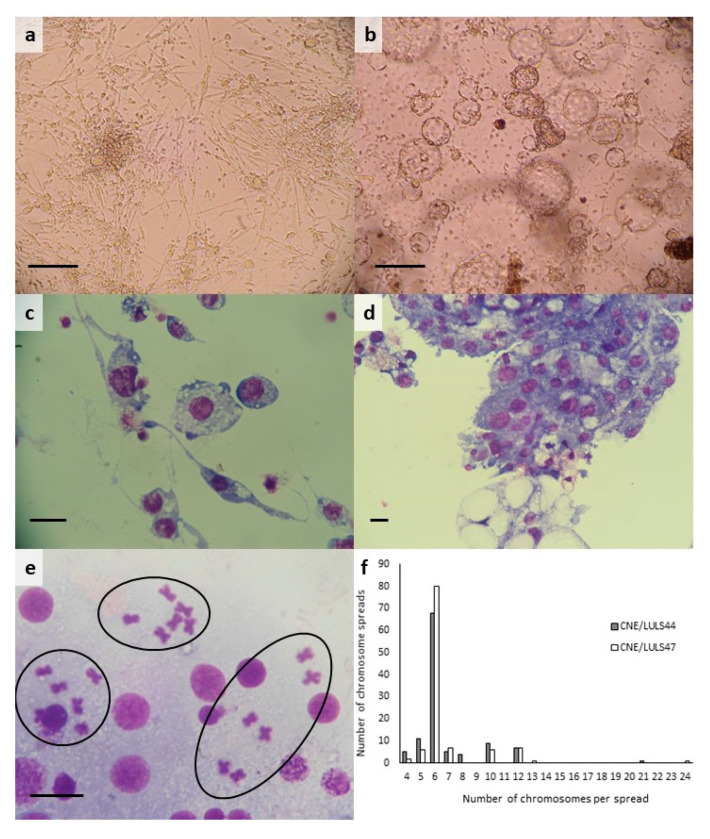
Light micrographs and karyotypes of *Culicoides nubeculosus* cell lines CNE/LULS44 and CNE/LULS47. (**a**) CNE/LULS44 cells at passage 15, 24 months after initiation, live, phase contrast, scale bar = 100 µm; (**b**) CNE/LULS47 cells at passage 3, 14 months after initiation, live, phase contrast, scale bar = 100 µm; (**c**) CNE/LULS44 cells at passage 13, Giemsa-stained cytocentrifuge smear, scale bar = 10 µm; (**d**) CNE/LULS47 cells at passage 5, Giemsa-stained cytocentrifuge smear, scale bar = 10 µm; (**e**) chromosome preparation from CNE/LULS47 cells at passage 6, showing three chromosome spreads (circled) with diploid number 2*n* = 6, Giemsa stain, scale bar = 10 µm; (**f**) distribution of chromosome numbers in 110 metaphase chromosome spreads each of CNE/LULS44 cells at passage 13 (grey bars) and CNE/LULS47 cells at passage 6 (white bars).

A total of 17 primary cultures were made from *C. nubeculosus* eggs supplied on three occasions between March and June 2018, using one or a combination (1:1) of the four culture media L-15, H-Lac, L-15B and Schneider’s. Of these, five cultures were lost to contamination because the egg batches contained some larvae that had hatched prior to surface sterilisation; larvae that hatched from intact eggs after surface sterilisation did not pose a contamination risk, but did consume embryonic tissues. A further 10 cultures did not contain viable embryonic tissues and were discontinued. Two primary cultures contained small clumps of viable tissues attached to the surface of the tube within 48 h. Some of these clumps contained contractile tissue that twitched or pulsated, and small, spindle-shaped or elongated cells began to migrate out from some of the clumps. This process continued slowly over the subsequent weeks, with a gradual increase in confluence and muscular activity. After 2–8 months, multicellular vesicles began to appear, either attached to the cell layer or free-floating individually or in clusters (Figure 1a,b).

The first successful primary culture, generated in L-15 medium from eggs laid in March 2018, was subcultured successfully after 6 months. Further subcultures followed and, when passage 5 was reached at 14 months, 4 tubes of cells were pooled and cryopreserved. Following resuscitation, the cells recovered rapidly, with attached cells visible after 24 h and the appearance of floating multicellular vesicles by 4 days. This cell line was designated CNE/LULS44 and had reached passage 16 at the time of writing. 

The second successful primary culture, initiated in L-15/H-Lac medium from eggs laid in June 2018, did not exhibit significant cell multiplication at first; the medium was changed to L-15/L-15B after 8 months in an attempt to stimulate cell growth. The primary culture was subcultured successfully after 10 months and cryopreserved and resuscitated successfully at passage 3, after 18 months. This cell line was designated CNE/LULS47, and had reached passage 9 at the time of writing. 

Both cell lines were confirmed as *C. nubeculosus* by amplification and sequencing of a fragment of the *cox1* gene; the resultant 616-617 bp sequences displayed high identity to eight *C. nubeculosus* sequences available at the time of writing. These included a sequence of unspecified geographical origin (GenBank accession no. KT974135.1; 99.68% identity; 100% query cover), and sequences from Turkey (GenBank accession no. MF594394.1: 100% identity; 70% query cover) and France (GenBank accession no. KF178273.1; 100% identity; 70% query cover). Five of the eight published *C. nubeculosus* sequences were only 472 bp in length which may explain why BLAST query coverage was lower (70%) for several of the matches to our 616–617 bp sequences. The pan-bacterial 16S rRNA PCR did not amplify any products from either cell line, and both tests for contaminating *Mycoplasma* gave negative results. 

### 3.2. Morphology and Karyotype of C. nubeculosus Cell Lines

Early passages of both *C. nubeculosus* cell lines comprised diverse populations of the attached round, spindle-shaped and elongated cells, cell clumps and floating multicellular vesicles derived from at least two different cell phenotypes. With time, and in later passages, elongated and clumped cells predominated in CNE/LULS44 (Figure 1a), while sheets of small, epithelial-like and spindle-shaped cells and floating vesicles predominated in CNE/LULS47 (Figure 1b). Giemsa-stained cytocentrifuge smears of CNE/LULS44 cells carried out at passage 13 revealed small round cells with dense blue cytoplasm, larger rounded cells with heavily-vacuolated cytoplasm, and spindle-shaped cells with long, threadlike terminal cytoplasmic protrusions (Figure 1c). Smears of CNE/LULS47 cells confirmed the presence of multicellular vesicles derived from at least two cell phenotypes: small cells with dense blue cytoplasm and larger cells with cytoplasm containing both small and extremely large vacuoles (Figure 1d). Karyotypes of CNE/LULS44 cells at passage 13 and CNE/LULS47 cells at passage 6 revealed that the expected diploid chromosome number 2*n* = 6 [41] predominated in both cell lines at, respectively, 62% and 73% of chromosome spreads counted (Figure 1e,f). Just over 6% of cells in both lines were tetraploid (4*n* = 12), while the remaining cells were aneuploid, with 4–21 and 4–24 chromosomes per spread from CNE/LULS 44 and CNE/LULS47 respectively (Figure 1f).

**Figure 2:**
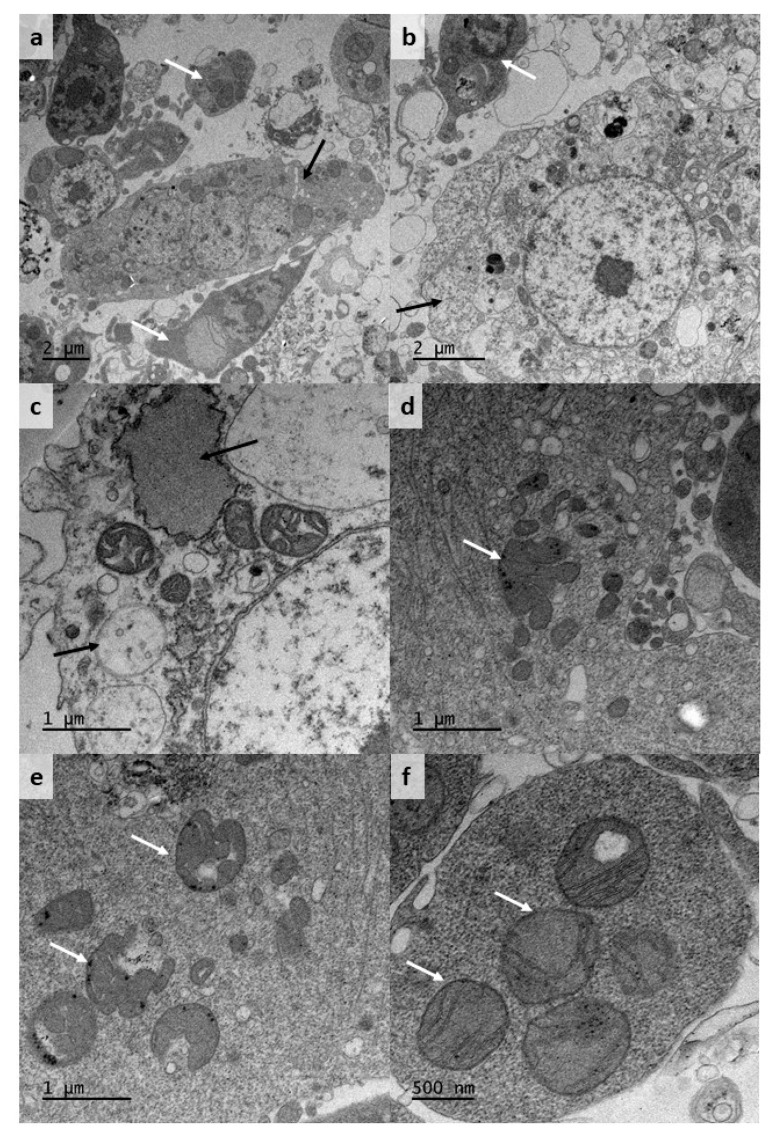
Ultrastructure of *Culicoides nubeculosus* cell line CNE/LULS44 at passage 11. (**a**,**b**) Low-magnification views of cells with relatively dense (white arrows) and relatively “open” (black arrows) cytoplasm; (**c**) part of a cell with “open” cytoplasm showing mitochondria and large electron-dense and electron-lucent vacuoles (arrows); (**d**) an apparently multilobed mitochondrion in an electron-dense cell (arrow); (**e**) bizarre lobed mitochondria (arrows) in an electron-dense cell; (**f**) large mitochondria with well-defined cristae (arrows) in an electron-dense cell.

TEM examination of CNE/LULS44 cells sampled at passage 11 revealed a diverse population with at least two predominant phenotypes (Figure 2a). Large cells, over 10 µm in diameter, had loose, relatively electron-lucent cytoplasm with a variety of vacuoles containing membranous or electron-dense material of unknown identity (Figure 2b) and mitochondria with few, well-defined cristae (Figure 2c). Smaller cells, up to ~5 µm in diameter, had more electron-dense and homogeneous cytoplasm containing small vesicles and relatively large, sometimes multilobed mitochondria (Figure 2d–f). Arrays of virus particles, previously reported in the *C. sonorensis* cell line CuVa [23], were not seen in any of the examined cells, and no other virus-like particles were observed.

### 3.3. Replication of BTV Serotype 1 in CNE/LULS44 Cells 

**Figure 3:**
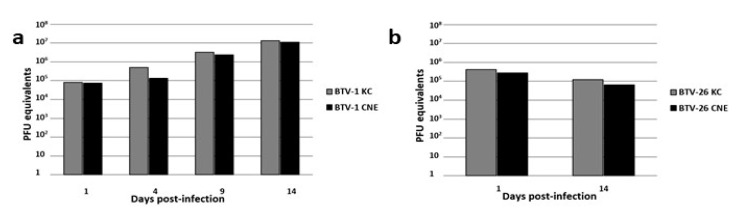
Levels of plaque-forming unit (PFU) equivalents of bluetongue virus serotypes BTV-1 and BTV-26 in *Culicoides nubeculosus* CNE/LULS44 (CNE) and *Culicoides sonorensis* KC cells over a 14-day period determined by real-time RT-PCR targeting BTV segment 10. (**a**) Increase in BTV-1 PFU equivalents in RNA extracted from pelleted CNE and KC cells; (**b**) decrease in BTV-26 PFU equivalents in RNA extracted from pelleted CNE and KC cells. Graphs show the results for pooled RNA extracted from triplicate wells.

Following inoculation with BTV-1, *C. nubeculosus* CNE/LULS44 cells and *C. sonorensis* KC cells supported similar levels of intracellular RNA replication (Figure 3a), with an increase of approximately 2–3 log_10_ PFU in CNE/LULS44 cells over the 14-day observation period. In contrast, a preliminary assay indicated that the levels of BTV-1 RNA detected in culture supernates were considerably higher in those of the KC cells compared to the CNE/LULS44 cells (data not shown). Following inoculation with BTV-26, no replication was detected by RT-PCR in CNE/LULS44 or KC cells (Figure 3b); by day 14 p.i, the number of PFU equivalents of BTV-26 were approximately fourfold lower in both cell lines.

## 4. Discussion

The first *Culicoides* cell lines, *C. sonorensis* CuVa and KC, were established 30 years ago from laboratory colony midge eggs [22,23]. Since then there has been only one published description of further attempts to establish *C. sonorensis* cell lines: eggs laid by field-collected midges yielded two cell lines (W3 and W8) [24]. Two additional unpublished lines and three clones derived from KC cells were listed subsequently [20]. Of these lines, only KC has been widely used in arbovirus research [25,26,27,28,29], while there has been one study using the W3 and W8 lines to propagate a mosquito-derived strain of the obligate intracellular bacterial symbiont *Wolbachia* [30]. We were unable to find any reports of attempts to generate cell lines from any other *Culicoides* species of either New or Old World origin. The two new *C. nubeculosus* cell lines reported here, CNE/LULS44 and CNE/LULS47, will therefore greatly increase the scope for research into many aspects of midges as vectors of viruses, protozoa and helminths, especially those relevant to Europe. They represent a novel comparative resource that is likely to expand research on *Culicoides* genetics and genomics and also provide an additional platform for the study of vector-host interactions. An important advantage is that long-term maintenance of both the cell lines and the *C. nubeculosus* colony from which they were derived is sustainable, ensuring the continued availability of matched resources. 

In the present study, the techniques applied to the generation of the *C. sonorensis* cell lines [22,24] were used as a starting point to develop an approach that was suited to *C. nubeculosus* eggs. The age of the eggs was important, as the presence of hatched larvae prior to surface-sterilisation invariably resulted in contamination of the cultures; hatching could be successfully delayed up to 5 days by holding eggs at 4 °C but not at 15 °C. A variation of the surface-sterilisation protocol applied to tick eggs [42], using 0.1% benzalkonium chloride followed by 70% ethanol, was found to be as efficient and harmless as the 70% ethanol followed by bleach used previously for *C. sonorensis* eggs [22]. Crushing the *C. nubeculosus* eggshells with a flattened glass rod was likely to be less damaging to the embryonic tissues than the glass tissue homogeniser used for *C. sonorensis* eggs [22], but it was probably less efficient as a proportion of eggs remained intact and hatched into larvae in the primary cultures. The most significant difference between the present study and previous reports was in the choice of culture medium: Schneider’s medium, routinely used for KC cells, was not found to be suitable for *C. nubeculosus* cells, while they grew prolifically in media based on L-15 (Leibovitz) medium. Interestingly, L-15 (Leibovitz) medium supplemented with 20% FBS, rather than Schneider’s medium, was used to propagate KC cells for karyotyping [41]. These alternative approaches may help generate cell lines from additional *Culicoides* species of veterinary or medical importance.

Morphologically, both *C. nubeculosus* cell lines are phenotypically heterogeneous, comprising round, spindle-shaped and elongated cells of different sizes, growing as attached individual cells, cells forming attached sheets and clumps, and floating multicellular vesicles. At the time of writing, the proportion of cells forming floating vesicles in CNE/LULS44 has greatly decreased, while the proportions of attached, sheet- and clump-forming cells remain constant, whereas vesicle-forming cells remain numerous in CNE/LULS47, making up at least half of the total biomass in the cultures. The phenotypic diversity is reflected in Giemsa-stained and TEM preparations, which demonstrate multiple cell types. In the TEM preparations, CNE/LULS44 cells displayed prominent mitochondria, some of which appeared to be multilobed or to have bizarre morphology. Little information on *Culicoides* mitochondria is available from published studies; numerous large mitochondria were reported in sheath cells surrounding mechanoreceptor sensillae of *C. nubeculosus* [43], and ovoid mitochondria with dense matrix and narrow cristae were described in *Culicoides punctatus* midgut cells [44], somewhat similar to those seen in the CNE/LULS44 cells with electron-dense cytoplasm. Variation in cell phenotype between *C. sonorensis* cell lines was also reported [24]; the two cell lines W3 and W8, derived from field-caught midges, differed from each other and the two cell lines CuVa and KC derived from laboratory colony midges. The absence of clearly-identifiable virus-like particles in CNE/LULS44 cells, in comparison to their presence in the CuVa cell line [23], does not necessarily mean that the *C. nubeculosus* cell lines do not harbour any endogenous viruses; it will be important to apply whole genome sequencing to both cell lines in future to explore their putative viromes [45].

There is very little published information on chromosome numbers in *Culicoides* spp. cells. The majority (68.5%) of *C. sonorensis* KC cells were reported to have a diploid number of 2*n* = 6 chromosomes; of the remainder, 30% were tetraploid and 1.5% had 24 chromosomes [41]. These values are broadly similar to those found for the two new *C. nubeculosus* cell lines in the present study, except that a much smaller proportion of spreads were tetraploid, and 32% and 20% of spreads were aneuploid in CNE/LULS44 and CNE/LULS47, respectively. Aneuploidy has frequently been reported in cell lines derived from other arthropod species including ticks [46], sand flies [47] and mosquitoes [48].

As BTV is one of the most economically important and widespread livestock viruses transmitted by *Culicoides*, we tested the ability of one of the novel *C. nubeculosus* cell lines to support replication of two serotypes: BTV-1, a typical serotype that is *Culicoides*-transmitted, and BTV-26, an atypical serotype that is suspected not to be transmitted by insects and failed to replicate in KC cells or *C. sonorensis* midges [49]. Using an established real-time RT-PCR assay, we detected replication of BTV-1 within CNE/LULS44 cells to a level comparable with that achieved in KC cells, derived from the North American BTV vector *C. sonorensis*. However, in contrast to these results for intracellular viral RNA, preliminary results indicated that extracellular viral RNA was present at lower levels in the supernates of CNE/LULS44 cultures than in those of KC cell cultures, which suggests the possibility that in CNE/LULS44 cells, a large proportion of virus particles remained cell-associated. Further studies using a wider range of midge-transmitted BTV serotypes are required to confirm and investigate this observation. In contrast, BTV-26 failed to replicate in both KC and CNE/LULS44 cells, confirming its status as an atypical bluetongue virus which appears to be unable to replicate in *Culicoides* midges. 

Vector competence of *C. nubeculosus* for BTV has been reported to be low, based on studies in which viral RNA replication was detected in only a small proportion of tested individuals following infection by membrane feeding on BTV-1-spiked horse blood [50]. Earlier studies, however, showed that *C. nubeculosus* was a competent experimental vector of BTV-4 following membrane feeding and supported virus replication following feeding on an infected sheep [51], and that co-infection with *O. cervicalis* microfilariae rendered a small proportion of individuals in a refractory *C. nubeculosus* population capable of transmitting an unspecified serotype of BTV [19]. The authors of the latter study speculated that the virus was able to enter the haemocoel of the insects via filarial-induced damage, thereby circumventing the midgut barrier, postulated to be the primary determinant of vector competence [52,53]. However, our results suggest that the situation within *C. nubeculosus* may be more complex if the apparent low ability of CNE/LULS44 cells *in vitro* to release mature BTV-1 particles reflects the conditions in intact midges. Despite broad assessments of susceptibility to infection and studies of barriers to arbovirus dissemination in *Culicoides* [52,53,54], BTV infection and release at the cellular level remains poorly explored. Further studies are needed to determine whether our results with BTV-1 are broadly representative of multiple BTV serotypes, whether the phenotypes represented in the CNE/LULS44 cell line include midgut cells (potentially limiting or blocking virion release), and whether manipulation of the culture conditions (temperature, pH, osmotic pressure) could facilitate the release of virus particles.

*C. nubeculosus* has been tested experimentally for the ability to support replication of, and/or transmit, other midge-borne arboviruses. Virus replication following intrathoracic inoculation, but not an oral infection, was demonstrated for the orbiviruses epizootic hemorrhagic disease virus, AHSV and Eubenangee virus, and the orthobunyavirus Akabane virus, all of which could be transmitted orally by *C. sonorensis* [55,56,57,58,59]. In contrast, oral infection of *C. nubeculosus* with the orthobunyavirus SBV was demonstrated experimentally [9], and transmission by *C. nubeculosus* following oral infection was demonstrated for two other orthobunyaviruses, Main Drain virus [60] and Shuni virus [61]. It will be interesting to test the ability of the two *C. nubeculosus* cell lines to support replication of other insect-borne orbiviruses, tick-borne orbiviruses, SBV and other orthobunyaviruses, and indeed intracellular bacteria of the genera *Wolbachia* [30], *Cardinium* [62,63] and *Rickettsia* [64], representatives of which have been reported to infect *Culicoides* biting midges.

In conclusion, the two new *C. nubeculosus* cell lines reported here will greatly expand the scope of research into *Culicoides*-borne pathogens at the cellular and molecular level. Virus replication and production can now be compared *in vitro* between cells of midge species with the different vectorial capacity for arboviruses such as BTV, facilitating a better understanding of the infection and release mechanisms underlying vector competence and virus transmission. The study not only provides a baseline for further characterisation of the existing *Culicoides* spp. cell lines, but will also facilitate the establishment of new cell lines from major arbovirus vector species for which only preliminary studies of colonisation have been carried out, such as the major Afrotropical BTV vector *C. imicola* [65]. The *C. nubeculosus* cell lines CNE/LULS44 and CNE/LULS47 are deposited in the Tick Cell Biobank at the University of Liverpool and will be available for distribution from December 2020.
